# A Dangerous Curiosity

**Published:** 1877-04

**Authors:** 


**A DANGEROUS CURIOSITY.**—It is the most natural thing in the world, when you have gone to bed, to get up, run to the window, hoist it and look out at an alarm of fire or any unusual noise or clamor going on outside. A lady was roused from her sleep by a cry of "Fire;" her chamber was as bright almost as day when she opened her eyes. She went to the window, and soon saw that it was her husband's cotton factory. She felt on the instant a shock at the pit of the stomach; the result was a painful disease which troubled her for the remainder of her life, a period of nearly fifteen years.

A young lady just budding into womanhood was called by the sound of midnight music to the window, and in her undress leaned her arm on the cold sill; the next day she had an attack of inflammation of the lungs which nearly killed her. She eventually recovered, only to be the victim of a life-long asthma, the horrible suffering from the oft-repeated attacks of which, during now these twenty years, is the painful penalty, to be paid over and over again as long as life lasts.

A letter just received from a successful banker, who has been an invalid for five years, every now and then spitting blood by the pint, with a harassing cough which makes every night and morning a purgatory, states that the immediate cause of all his sufferings, and the final blasting of life's prospects, was his getting up on a cool night, to look out of his chamber-window, his body being in a perspiration at the time. That sturdy old Trojan, Dr. Johnson, used to say that "mankind did not so much require instructing as reminding;" hence the present reminder, that it is dangerous for people to be poking their nightcaps out of window after night-fall. Another mischievous habit in the same direction, may have pertinent mention here: standing in the street doorway in cold weather, while the door itself is open, in taking leave of visitors. The cold air from without rushes into the dwelling, causing a draught, which chills the whole body almost instantly. It is a hundred times safer to close the door and stand without, bare-headed. Many a tedious case of sickness and suffering has been occasioned, and even life itself has been lost, by an exposure, apparently so trifling. May our readers remember these things, and teach them to their children on the instant.

